# Reward sensitivity deficits modulated by dopamine are associated with apathy in Parkinson’s disease

**DOI:** 10.1093/brain/aww188

**Published:** 2016-07-24

**Authors:** Kinan Muhammed, Sanjay Manohar, Michael Ben Yehuda, Trevor T.-J. Chong, George Tofaris, Graham Lennox, Marko Bogdanovic, Michele Hu, Masud Husain

**Affiliations:** ^1^ 1 Nuffield Department of Clinical Neurosciences, University of Oxford, Oxford, UK; ^2^ 2 Department of Experimental Psychology, University of Oxford, Oxford, UK; ^3^ 3 Department of Cognitive Science, Macquarie University, Sydney, Australia

**Keywords:** apathy, vision, Parkinson’s disease, drug treatment, neuropsychology

## Abstract

Apathy is a debilitating and under-recognized condition that has a significant impact in many neurodegenerative disorders. In Parkinson’s disease, it is now known to contribute to worse outcomes and a reduced quality of life for patients and carers, adding to health costs and extending disease burden. However, despite its clinical importance, there remains limited understanding of mechanisms underlying apathy. Here we investigated if insensitivity to reward might be a contributory factor and examined how this relates to severity of clinical symptoms. To do this we created novel ocular measures that indexed motivation level using pupillary and saccadic response to monetary incentives, allowing reward sensitivity to be evaluated objectively. This approach was tested in 40 patients with Parkinson’s disease, 31 elderly age-matched control participants and 20 young healthy volunteers. Thirty patients were examined ON and OFF their dopaminergic medication in two counterbalanced sessions, so that the effect of dopamine on reward sensitivity could be assessed. Pupillary dilation to increasing levels of monetary reward on offer provided quantifiable metrics of motivation in healthy subjects as well as patients. Moreover, pupillary reward sensitivity declined with age. In Parkinson’s disease, reduced pupillary modulation by incentives was predictive of apathy severity, and independent of motor impairment and autonomic dysfunction as assessed using overnight heart rate variability measures. Reward sensitivity was further modulated by dopaminergic state, with blunted sensitivity when patients were OFF dopaminergic drugs, both in pupillary response and saccadic peak velocity response to reward. These findings suggest that reward insensitivity may be a contributory mechanism to apathy and provide potential new clinical measures for improved diagnosis and monitoring of apathy.

## Teaser

Apathy is extremely common in neurodegenerative disorders such as Parkinson’s disease. Muhammed *et al.* report that lack of sensitivity to rewards may underlie apathy, with dopamine playing a modulatory role. The study provides a basis for objective clinical markers of motivation and treatment efficacy in neurodegenerative conditions.

## Graphical abstract

**Figure:**
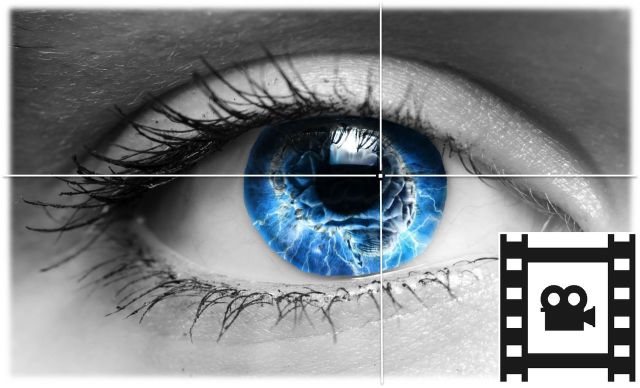
Apathy is extremely common in neurodegenerative disorders such as Parkinson’s disease. Muhammed *et al.* report that lack of sensitivity to rewards may underlie apathy, with dopamine playing a modulatory role. The study provides a basis for objective clinical markers of motivation and treatment efficacy in neurodegenerative conditions.

## Introduction

Apathy is a debilitating disorder of motivation for goal-directed behaviour ( Marin, 1991 ; Cummings *et al.* , 1994 ; Stuss *et al.* , 2000 ; Robert *et al.* , 2002 ; Levy and Dubois, 2006 ; Starkstein and Leentjens, 2008 ). It has distinct and dissociable attributes from other neuropsychiatric disorders such as depression ( Starkstein *et al.* , 1992 ; Kirsch-Darrow *et al.* , 2011 ; Jordan *et al.* , 2013 ) and has been categorized on the basis of either reduced self-initiated motor actions—referred to as a deficit in auto-activation ( Levy and Dubois, 2006 ); blunted cognitive inquisitiveness; or emotional indifference ( Stuss *et al.* , 2000 ). It is common in neurodegenerative disorders such as Alzheimer’s disease ( Landes *et al.* , 2001 ), small vessel cerebrovascular disease and vascular dementia ( Hollocks *et al.* , 2015 ), frontotemporal dementia and amyotrophic lateral sclerosis ( Lillo *et al.* , 2012 ; Mioshi *et al.* , 2014 ; Bertoux *et al.* , 2015 ). It is also common in Parkinson’s disease, affecting up to 70% of patients ( Starkstein *et al.* , 1992 ; Pluck and Brown 2002 ; van Reekum *et al.* , 2005 ; Dujardin *et al.* , 2007 ; Aarsland *et al.* , 2009 ; Ahearn *et al.* , 2012 ; Cubo *et al.* , 2012 ; Santangelo *et al.* , 2013 ; Sinha *et al.* , 2013 ; den Brok *et al.* , 2015 ), including approximately a quarter of drug naïve cases ( Pedersen *et al.* , 2010 ). Clinical apathy is defined as impairment in at least two of the three dimensions of apathy ( Mulin *et al.* , 2011 ).

Non-motor symptoms such as apathy have a significant impact in Parkinson’s disease ( Martinez-Martin *et al.* , 2011 ; Duncan *et al.* , 2014 ; Muhammed and Husain, 2016 ). Motivational deficits are one of the major determinants of quality of life ( Barone *et al.* , 2009 ; Benito-León *et al.* , 2012 ), often preceding the onset of motor disturbances ( Pont-Sunyer *et al.* , 2015 ) and unrelated to physical disability ( Pluck and Brown, 2002 ). Although we have begun to understand the extent of the problem faced by those suffering apathy and its deleterious effects on everyday function ( den Brok *et al.* , 2015 ; Pagonabarraga *et al.* , 2015 ), we still understand little about potential underlying mechanisms ( Marin, 1996 ; Dujardin and Defebvre, 2012 ; Sinha *et al.* , 2013 ).

A few recent studies have suggested that reward sensitivity might be an important component of apathy in health and disease ( Czernecki *et al.* , 2002 ; Adam *et al.* , 2013 ; Bonnelle *et al.* , 2015 *a* , *b* ). Investigations in both monkeys and humans show that a simple way to measure such sensitivity is to use eye movement and pupillary indices ( Hong and Hikosaka, 2008 ; Chen *et al.* , 2014 ; Rudebeck *et al.* , 2014 ; Manohar and Husain, 2015 ; Manohar *et al.* , 2015 ). We developed a novel eye-tracking paradigm to index reward sensitivity in Parkinson’s disease using saccadic and pupillary properties and sought to determine if dysfunctional reward processing contributes to clinical apathy. To date, few have attempted to explore this hypothesis. Most work has quantified apathy using questionnaire and clinical interview measures, relating scores to other disease states including depression ( Kirsch-Darrow *et al.* , 2011 ) and dementia ( Dujardin *et al.* , 2009 ), or linking outcomes to structural and functional imaging findings ( Reijnders *et al.* , 2010 ; Carriere *et al.* , 2014 ; Baggio *et al.* , 2015 ).

Previously, paradigms used to assess reward-based decision-making, including gambling and effort-based tasks, have demonstrated deficits in Parkinson’s disease ( Czernecki *et al.* , 2002 ; Cools *et al.* , 2003 ; Schmidt *et al.* , 2008 ; Torta and Castelli, 2008 ; Torta *et al.* , 2009 ; Chong *et al.* , 2015 ), but only one has reported the relationship to apathy ( Martinez-Horta *et al.* , 2014 ). Although informative, these tasks cannot dynamically measure reward-related processes that are likely to be active during the evaluation and execution stage of the decision itself. Galvanic skin responses have been used as a proxy for reward sensitivity and value encoding ( Schmidt *et al.* , 2008 ); however, it remains to be determined how this might relate specifically to apathy in Parkinson’s disease. Other studies have demonstrated deficits in reward-based learning in Parkinson’s disease, particularly on reversal learning ( Swainson *et al.* , 2000 ; Czernecki *et al.* , 2002 ; Frank *et al.* , 2004 ; Cools *et al.* , 2006 ; Peterson *et al.* , 2009 ; Schmidt *et al.* , 2014 ; Sharp *et al.* , 2016 ). Performance on such tasks, however, is likely to rely on several cognitive processes, of which reward sensitivity might be only one. Moreover, these investigations either did not examine the relationship to clinical apathy or did not have a sufficient sample to make any inferences on this issue.

Frontrostriatal dysfunction and dopamine depletion in brain regions implicated in motivation such as the ventral striatum, ventral tegmental area and substantia nigra pars compacta ( Robbins and Everitt, 1996 ; Levy and Dubois, 2006 ; Drui *et al.* , 2013 ; Manohar and Husain, 2016 ) make Parkinson’s disease a unique model to study apathy. Dopamine modulates a number of mechanisms including vigour, reward prediction and initiation of effortful behaviour ( Schultz, 2007 ; Kurniawan *et al.* , 2011 ; du Hoffmann and Nicola, 2014 ; Chong *et al.* , 2015 ). However, based on clinical evidence, it is likely also to exhibit a modulatory function in apathy. For example, patients with apathy secondary to basal ganglia infarcts show improvement when treated with dopamine ( Laplane *et al.* , 1989 ; Schmidt *et al.* , 2008 ; Adam *et al.* , 2013 ) and Parkinson’s disease patients with subthalamic nucleus deep brain stimulation (STN-DBS) can develop apathy when their dopaminergic drug dose is reduced postoperatively ( Drapier *et al.* , 2006 ; Czernecki *et al.* , 2008 ; Thobois *et al.* , 2013 ). Yet the exact processes that dopamine influences to alter motivation in these patient groups remains to be fully characterized.

In this study we investigated whether reduced reward sensitivity might be a key mechanism underlying apathy in Parkinson’s disease. Does insensitivity to rewards cause subjective devaluation of incentives, thereby contributing to the behavioural phenotype of apathy? We hypothesized that pupillary dilation and invigoration of actions would be increased for larger rewards, that this would be dependent on dopamine and, crucially, blunted in apathetic patients. Using a simple novel monetary incentivized eye-tracking paradigm, pupillary responses and oculomotor measures of reward sensitivity were recorded. This protocol was conducted on patients with Parkinson’s disease, healthy age-matched controls and young healthy controls, and the relationship to apathy examined. Thirty patients were tested both ON and OFF dopamine medication in two counterbalanced sessions. To control for autonomic dysfunction effects on pupillary response to reward, patients with Parkinson’s disease also wore a portable single lead ECG monitor while at rest overnight to measure heart variability, which is an important index of autonomic function ( Acharya *et al.* , 2006 ).

## Materials and methods

### Participants

The study was approved by the local ethics committee and written consent was obtained from all subjects in accordance with the Declaration of Helsinki. Participants were informed that they would be paid according to task performance, a minimum of £8 and maximum of £12 was paid and transport costs were reimbursed. Patients and elderly participants were assessed for apathy using the Lille Apathy Rating Scale (LARS), which has been validated in Parkinson’s disease ( Sockeel, 2006); they were screened for depression with the Beck Depression Inventory-II (BDI-II; Beck *et al.* , 1996 ) and for cognitive impairment on the Montreal Cognitive Assessment (MoCA) ( Nasreddine *et al.* , 2005 ). The severity of Parkinson’s disease was measured using the Movement Disorder Society Unified Parkinson’s Disease Rating Scale (UPDRS) ( Goetz *et al.* , 2008 ). No participant had a history of psychiatric illnesses or autonomic dysfunction.

#### Parkinson’s disease demographics

Forty patients with Parkinson’s disease were included in the study, 26 patients were male, and 34 right-handed. All had a clinical diagnosis of idiopathic Parkinson’s disease and no history of other major neurological or psychiatric illnesses. They were recruited from clinics in the Oxfordshire area. Ten were drug-naive and 30 were currently established on levodopa and dopamine agonist combinations. Nine patients were on levodopa only, two on dopamine agonists only and 19 on a combination of both. The 30 patients ON medication were tested in two counter-balanced morning sessions, one session having taken their dopaminergic medication as normal (ON) and the other after overnight withdrawal (OFF).

Using the clinical interview LARS, a score of ≥−22 was set as the cut-off for apathy, encompassing mild to severe apathy symptoms ( Sockeel, 2006 ). Non-drug naive patients were divided into two groups; those suffering with apathy ( *n =* 14) mean LARS −15.3, standard deviation (SD) 6.7; and those without ( *n =* 16) mean LARS −28.5, SD 2.8. Drug naïve patients were also divided based on their LARS score and included in the full hierarchical analysis with the Parkinson’s disease patients who were OFF, half were allocated to each apathy and non-apathy groups taking the total to 19 and 21, respectively. On breakdown of the LARS, the apathetic patients scored significantly worse than the non-apathetic patients in all three apathy domains. This included behavioural apathy (action initiation), cognitive apathy (intellectual curiosity) and emotional apathy ( *P <* 0.01). They did not, however, show any differences in the additional self-awareness component of the LARS.

There were no significant differences between apathetic and non-apathetic patients in age, motor severity (UPDRS part III), cognitive impairment, depression scores or levodopa equivalent dose **(**Table 1**)** . On breakdown of the UPDRS components, apathetic patients had significantly worse non-motor symptoms (UPDRS part I). This difference was driven by a lower score on the apathy assessment question ( *P <* 0.05). Disease duration was significantly longer in the non-apathetic patient group and their average age at diagnosis trended towards an earlier onset but there were no correlations with apathy level (LARS) and age in Parkinson’s disease r _s_ = 0.045, *P* = 0.813. None of the patients had a current diagnosis or previous history of impulse control disorder.

**Table 1:** Demographics

	Healthy elderly controls	Parkinson’s disease (medicated)	Controls versus Parkinson’s disease	Non-apathetic patients	Apathetic patients	Non-apathy versus apathy	Parkinson’s disease (drug naïve)
			*P* -value	(LARS < –22)	(LARS ≥ –22)	*P* -value	
*n*	31	30		16 ON/OFF	14 ON/OFF		10 (5 apathetic)
Age (years)	67.5 (±8.5)	66.5 (±5.9)	0.6	65.9 (±5.6)	67.3 (±6.4)	0.52	65.8 (±6.3)
Apathy (LARS)	−29 (±4.3)	−22.3 (±8.3)	<0.0001*	−28.5 (±2.8)	−15.3 (±6.7)	<0.0001*	−22.5 (±7.9)
Depression score (BDI)	4.9 (±5)	13.3 (±6.8)	<0.0001*	11.7 (±5.5)	15.2 (±7.8)	0.16	12.2 (±8.6)
MoCA	28 (±1.5)	28 (±1.9)	0.5	28.2 (±1.6)	27.8 (±2.3)	0.57	26.1 (±3.3)
UPDRS I	n/a	8 (±4.8)	n/a	6.1 (±3.9)	10.1 (±4.9)	0.02*	7.8 (±5.9)
UPDRS III ON	n/a	19.4 (±9.8)	n/a	18.4 (±2.6)	20.4 (±2.4)	0.59	24.4 (±9.3)
Hoehn and Yahr Stage	n/a	1.3 (±0.7)	n/a	1.4 (±0.6)	1.2 (±0.8)	0.54	1.4 (±1.4)
Disease duration (years)	n/a	5.0 (±4)	n/a	6.8 (±4.6)	3.2 (±2.2)	0.02*	1.1 (±1.5)
Average age at diagnosis (years)	n/a	61.4 (±7.5)	n/a	59.2 (±7.6)	64 (±6.7)	0.08	64.9 (±6.6)
Hours since last dose ON versus OFF	n/a	2.5 (±2.2) versus 14.2 (±4)	n/a	2.1 (±1.1) versus 15.2 (±3.9)	2.9 (±3) versus 13.1 (±4)	0.32	n/a
						0.24	
Levodopa equivalent dose (mg/24 h)	n/a	507 (±61.2)	n/a	493.9 (±75.8)	522.7 (±101.4)	0.82	n/a

Demographics of healthy elderly age matched controls, total patients with Parkinson’s disease, drug-naïve patients with Parkinson’s disease, and patients divided into apathetic and non-apathetic subgroups. Numbers in brackets represent standard deviations and standard error of the mean where appropriate. *Significant result.

MoCA = Montreal Cognitive Assessment; BDI = Beck Depression Inventory II.

#### Control participants demographics

Thirty-one age matched healthy participants were tested in a single session; 13 were male and 27 right-handed. A further 20 young participants were also examined (average age 25.75 years, SD 4.53, 19 right-handed, eight male). The control subjects were recruited from a volunteer database and were also screened for any history of neurological or psychiatric conditions. All participants had normal, or corrected to normal vision at the time of testing. There were no differences in age between the patients and elderly control participants or any differences in cognitive ability on the MoCA. Patients with Parkinson’s disease had significantly worse total LARS scores than elderly controls and also higher BDI scores; however, average depression scores in both groups were not classified as depressed and did not exceed mild mood disturbance/borderline depression (cut-off >20). Only one elderly control participant fell in the apathetic range, with a LARS score of −15. Parkinson’s disease patients and elderly controls also had MoCA scores in the normal range ( Table 1**)** .

### Experimental paradigm

**Figure 1:**
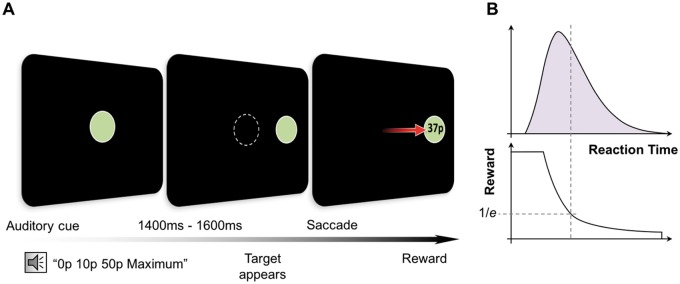
**Experimental paradigm.** ( **A** ) Participants heard a recorded auditory cue that informed them of the maximum reward available for each trial: ‘0p/10p/50p maximum’. After a randomly variable fore-period of 1400, 1500 or 1600 ms the central fixation disc disappeared and concurrently a new target disc appeared. Participants were rewarded according to reaction time, with the reward obtained displayed within the target disc in pence (e.g. 37p). ( **B** ) The absolute amount of reward earned varied with reaction time, but crucially was dynamically adjusted according to mean reaction time of each participant at any point during the experiment. To calculate the reward obtained for each trial, an adaptive exponential fall-off based on the mean reaction time of the preceding 20 trials was used. Participants received a proportion of the maximum amount on offer dependent on performance. This adaptive procedure allowed difficulty level to be maintained consistently over the experiment and importantly provided equal overall reward amounts to all participants. Thus it was not the case that apathetic patients earned less as they proceeded through the task nor did they have less external incentivization.

An infrared eye tracker (Eyelink 1000, SR Research) was used to monitor pupil diameter and eye position. Participants were instructed that the task involved making quick eye movements, the faster they looked at a target, the greater the proportion of reward on offer they would obtain ( Fig. 1 A). Each trial commenced with the onset of a disc at screen centre. After they had fixated this for 500 ms, participants heard a recorded voice that informed them of the maximum reward available for that particular trial: ‘0p/10p/50p maximum’. Subsequently, after a randomly variable period of 1400, 1500 or 1600 ms the central fixation disc disappeared and concurrently a new target disc appeared randomly either to the left or right. The targets measured 4° in diameter and appeared at 11° eccentricity.

Participants received a proportion of the maximum reward on offer dependent on performance. The absolute amount of reward earned varied with reaction time, but crucially was dynamically adjusted using an ‘adaptive’ exponential fall-off based on average reaction time of the preceding 20 trials. This allowed difficulty level to be maintained consistently over the experiment, factored in different individual fatigue rates and baseline reaction times and importantly provided equal reward amounts to all participants ( Fig. 1 B). Thus any differences in performance between groups could not be attributed to differences in overall rewards earned; see Supplementary material for further details. To ensure participants were equally extrinsically motivated by the amount of reward they earned, feedback was displayed within the target disc in pence at the end of each trial.

Disc luminance was matched across all trials so as not to affect pupil dilation. If the reward obtained was 10p (pence) or more an audible bell sound was also played, and if greater than 30p a ‘cash register’ sound was played after reward delivery at the end of the trial; no sound was heard for less money. Participants performed five blocks of 54 trials each, with a 5-min break between the first three and last two blocks, resulting in 270 trials in total with 90 trials for each of the three reward levels. Behavioural indicators of task performance included saccadic speed and saccadic variability. In debriefing, all participants said that they had attempted to maximize their gains, although people varied in strategy with some saying they tried to speed up for bigger rewards, while others said they tried to slow down on no reward trials.

### Eye tracker data recording

Eye tracking was performed in a dimly lit room 60 cm in front of a 21” CRT (1024 × 768 pixels; 100 Hz refresh). Stimuli were presented on a Windows computer running Matlab (The MathWorks) and Psychophysics Toolbox. The frame-mounted infrared tracker monitored left eye position and sampled at 1 kHz. Eye movements were measured online by the Eyelink computer and transferred directly to the presentation computer to provide immediate feedback. Nine-point calibration was performed. Eye movement analysis was carried out using custom-made Matlab code. An eye movement was classified as a saccade if it was >2°, and the accepted landing area to register completion of the saccade was 5° in radius from target centre. The first landing point within the target area was used to classify saccade completion. Reaction time was calculated from target onset to the time when a saccade was registered as complete, and peak saccadic velocity computed as maximum velocity recorded during the saccade. Pupil dilation was calculated as a proportional change from average baseline pupil size measured in Eyelink units. Recordings were time locked to the reward cue onset and normalized using a 200 ms baseline subtraction for each trial. Pupil traces lost due to blinks were interpolated. A moving average smoothing window of 100 ms was applied to the final recordings.

### Eye tracker data analysis

Pupillometry analysis was performed using split-level ANOVA, with within-subjects factors of Drug (ON, OFF) and Reward (0p, 10p, 50p), and between-subjects factor of Apathy (apathetic, non-apathetic). To account for any non-sphericity in the data, where appropriate, statistics are reported with Greenhouse-Geisser correction. Bonferroni corrected pairwise comparisons were made if significant interactions were present. Significance was taken as *P* -values of <0.05. Correlations with questionnaires were performed using Spearman rank non-parametric testing and Pearson correlations were used for parametric behavioural outcome comparisons. Statistics were completed using Matlab and SPSS.

### Heart rate variability recording and analysis

All patients with Parkinson’s disease on the day of carrying out the eye tracking experiment also wore a portable Actiwave Cardio single channel ECG recorder and tri-axis accelerometer (CamNtech Ltd). This was placed on the mid-chest using adhesive electrodes and worn overnight while sleeping. Recordings were sampled at 128 Hz and a 30-min epoch for each patient between the hours of 1 am and 4 am was selected when completely at rest, as detected by no accelerometer activity. Heart rate variability analysis was performed using time domain and frequency domain methods as performed by other studies of heart rate variability in Parkinson’s disease ( Haapaniemi *et al.* , 2001 ). Heart rate variability data were not normally distributed, therefore comparisons between apathetic and non-apathetic patients with Parkinson’s disease were made using non-parametric Mann Whitney-U tests.

## Results

### Pupillometry

Pupillary modulation by reward was taken as mean proportional change in pupil size from baseline pupil diameter at the beginning of each trial, time locked to the onset of the auditory reward cue. Mean pupillary size from 1400–2400 ms after the auditory cue was used as the epoch of interest. Our question here is whether pupil size varied according to the auditory reward cue in each trial. An individual’s ‘pupillary reward sensitivity’ was defined as the difference in mean proportional pupil change between the maximal 50p reward and 0p reward conditions, over the time epoch defined above. A larger difference indicated greater reward sensitivity.

#### Reward sensitivity declines with age

**Figure 2:**
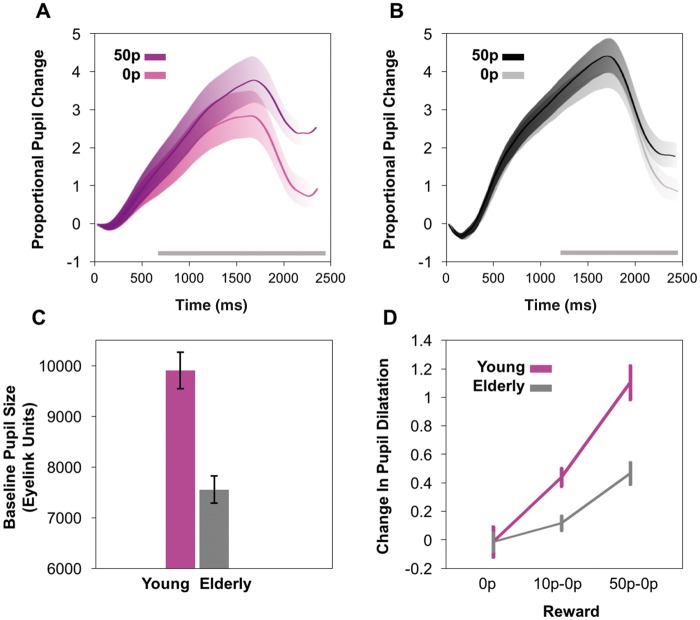
**Pupillary responses in control participants.** ( **A** ) Mean pupillary trace in young control participants after onset of reward cue at time 0 ms to end of trial. Pupil dilation was measured as proportional change from baseline prior to stimuli onset. Greater proportional change was observed for larger rewards compared to 0p reward. A significant difference between the 50p maximal reward (dark purple) and 0p reward level (light purple) was present from ∼630 ms to the end of the trial ( *P* < 0.05), denoted by grey bar at bottom of plot. Shaded areas represent standard errors of the mean (SEM). ( **B** ) Mean pupillary trace in elderly control participants after onset of reward cue. A significant difference between 50p maximal reward (black) and 0p reward (grey) was present from ∼1200 ms to the end of the trial ( *P* < 0.05), denoted by grey bar at bottom of plot. ( **C** ) Mean pupil baseline size as taken at the start of each trial and measured using Eyelink arbitrary units (A.U). Young controls had significantly larger baseline pupil size compared to elderly controls. ( **D** ) Proportional pupillary change as a function of reward level in young compared to elderly controls, taken as mean pupil dilation between 1400–2400 ms. Plots have been normalized to the 0p baseline to demonstrate the reward sensitivity slopes between young (purple) and elderly control participants (grey). Although both groups demonstrated greater pupillary dilation with increasing reward magnitude, there was reduced pupil reward sensitivity in elderly compared to young participants despite elderly controls having a smaller baseline pupil size and therefore more capacity to dilate for rewards.

In young and elderly controls, pupillary dilation was significantly increased by the magnitude of monetary reward on offer ( Fig. 2 A and B). As mentioned previously, reward sensitivity was defined as the difference in pupil response between the maximal 50p reward on offer and the 0p reward. Repeated measures ANOVA in the young controls over the time epoch of interest demonstrated a significant main effect of reward [ *F* (1.5,28.2) = 20.2, *P <* 0.001] and *post hoc* comparisons between rewards (0p versus 10p, 10p versus 50p and 0p versus 50p) revealed significant differences in pupillary response between each level ( *P <* 0.01). In elderly controls there was also a main effect of reward, [ *F* (1.7,49.6) = 9.0, *P <* 0.001]. However, the extent of reward sensitivity was not as great, as conveyed by the shallower slope of the pupil size between 0p and 50p against reward on offer ( Fig. 2 D). In the elderly, *post hoc* comparisons showed a change in pupillary size between each reward at the *P <* 0.01 significance level, except between 0p and 10p where no significant difference was found. The reduction in reward sensitivity with age was reiterated when comparing directly between young and elderly controls. Here, there was no significant overall main effect of group demonstrated [ *F* (1,49) = 1.1, *P =* 0.3]. However, a significant main effect of reward was still evident [ *F* (1.6,78.6) = 30.8, *P <* 0.001] and crucially there was also a significant reward by group interaction, so the extent of pupillary response to reward for increasing reward magnitudes was reduced dependent on increasing age group, *F* (1.6,49) = 4.8, *P <* 0.02. These findings were evident despite young controls having significantly larger mean baseline pupil size compared to elderly controls and therefore less capacity to dilate for reward ( Fig. 2 C). Although elderly participants had on average smaller pupils compared to young people, and therefore more scope for proportional change, their pupillary response did not modulate with reward to the same degree, i.e. they had less change in pupil size between the 0p and 50p conditions.

#### Dopaminergic medication increases reward sensitivity in Parkinson’s disease

**Figure 3:**
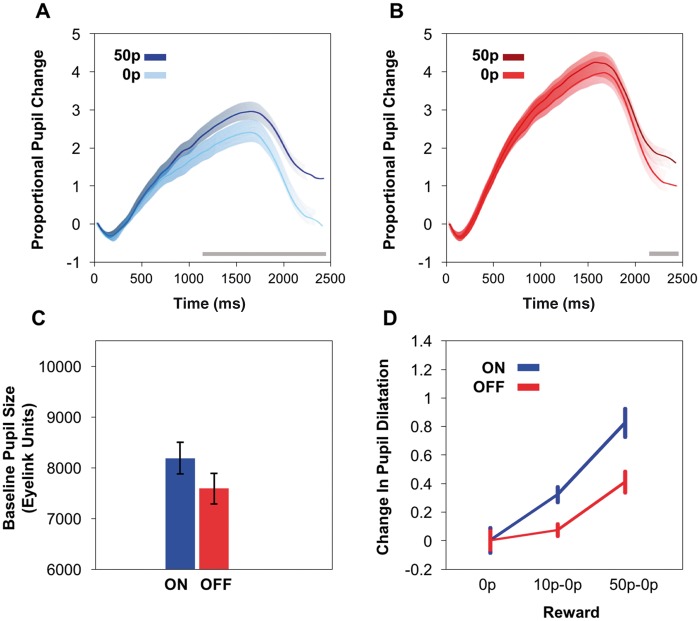
**Pupillary responses in patients.** ( **A** ) Mean pupillary trace in Parkinson’s disease patients ON dopaminergic medication. Greater proportional change was observed for the larger reward, with a significant difference between 50p maximal reward (dark blue) and 0p reward level (light blue) present from ∼1100 ms ( *P* < 0.05), denoted by grey bar at bottom of plot. ( **B** ) Mean pupillary trace in Parkinson’s disease OFF dopaminergic medication after onset of reward cue. A significant difference between the 50p maximal reward (dark red) and 0p reward (light red) was observed but only from ∼2100 ms onwards ( *P* < 0.05), denoted by grey bar at bottom of plot. This differential effect of reward was significantly delayed in onset compared to when ON dopaminergic medication. Moreover, the difference in response to the two reward levels was less in the OFF drug state. ( **C** ) Mean pupil baseline size as taken at the start of each trial and measured using Eyelink arbitrary units (A.U). Parkinson’s disease ON had significantly larger baseline pupil size compared to Parkinson’s disease OFF. ( **D** ) Proportional pupillary change as a function of reward level in Parkinson’s disease ON (blue) compared to Parkinson’s disease OFF (red) dopaminergic medication, taken as the mean pupil dilation between 1400–2400 ms. Changes have been normalized to the 0p baseline to demonstrate the relationship between reward sensitivity slopes. There was a reduced reward sensitivity in the OFF drug state compared to ON, despite increased scope for dilation when OFF due to smaller baseline pupil sizes.

Similar to controls, there was a main effect of reward on pupil size in the Parkinson’s disease group, with larger rewards on offer eliciting greater pupillary dilation over time **(**Fig. 3 A and B). A main effect of drug state was also present, revealing Parkinson’s disease patients ON dopamine had greater pupillary response to rewards compared to when OFF [ Fig. 3 D; main effect of drug state *F* (1,28) = 17.6, *P <* 0.001; main effect of reward *F* (1.4,39.5) = 15.0, *P <* 0.0001]. There was also a significant interaction between drug state and reward, with reduced reward sensitivity when OFF dopaminergic medication as indicated by a shallower sensitivity slope, [ *F* (1.9,53.8) = 5.7, *P <* 0.01]. *Post hoc* pairwise comparisons emphasised this further by revealing significantly larger pupillary changes for increasing reward levels when ON dopamine (0p versus 10p, *P <* 0.01; 10p versus 50p, *P <* 0.001; 10p versus 50p, *P <* 0.0001). However, this was blunted in the OFF state, with significant differences only between 0p versus 50p ( *P <* 0.01) and 10p versus 50p ( *P <* 0.01) and not between 0p versus 10p.

To ensure that age differences in the Parkinson’s disease group did not affect pupil reward sensitivity, a correlation analysis was performed. No significant correlation was found in either drug state between pupil reward sensitivity and age, ON r = −0.31, *P =* 0.1, and OFF r = −0.24, *P =* 0.2. There was also no significant difference in baseline pupil size between each of the reward levels prior to incentives being offered. This is an important observation as it means baseline pupil size did not influence the changes in pupil dilation for different reward levels. This was true both in the ON and OFF drug state with no significant drug state by reward interaction for baseline pupil size.

There was, however, a significant difference in baseline pupil size between drug states. Parkinson’s disease patients ON had larger pupils at baseline compared to those OFF (main effect of drug state [ *F* (1,29) = 20.7, *P <* 0.001; Fig. 3 C]. This further strengthens the hypothesis that dopamine increases reward sensitivity. When ON dopamine, the pupil was more dilated at baseline, implying less scope for subsequent pupillary dilation for reward compared to the OFF state in which the pupil had greater capacity to enlarge. Contrary to this, we actually found that larger rewards on offer lead to greater proportional pupil dilation in the ON state compared to the OFF state ( Fig. 3 D **)** . Therefore, despite the changes dopamine has on baseline pupil size, it still led to increased pupillary reward sensitivity (difference in dilation between the 0p and 50p condition). There was no significant difference in baseline pupil size between Parkinson’s disease patients ON and elderly controls, or Parkinson’s disease patients OFF and elderly controls.

#### Reward sensitivity is blunted in apathetic patients with Parkinson’s disease

**Figure 4:**
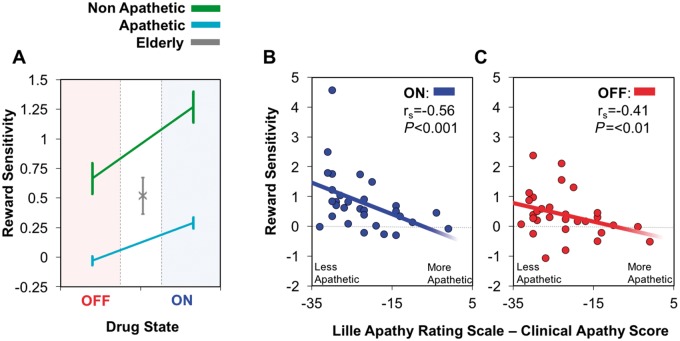
**Pupillary responses in apathetic vs non-apathetic Parkinson’s disease cases.** ( **A** ) Mean pupil reward sensitivity over 1400–2400 ms in apathetic Parkinson’s disease patients (cyan) was significantly reduced compared to non-apathetic patients (green) both ON and OFF dopaminergic medication. There was an increase in reward sensitivity in both apathetic and non-apathetic patients when ON dopamine, but no significant interaction between the drug state and apathy level. Comparison of average pupil reward sensitivity between apathetic and non-apathetic Parkinson’s disease patients to elderly controls (grey) showed a significant reduction in reward sensitivity in apathetic Parkinson’s disease patients when OFF dopamine and greater reward sensitivity in non-apathetic Parkinson’s disease patients when ON. ( **B** ) A significant correlation between average pupil reward sensitivity in Parkinson’s disease patients ON and their clinical interview LARS score. More apathetic patients exhibit less pupillary reward sensitivity compared to more motivated patients. ( **C** ) A significant correlation between pupil reward sensitivity and clinical interview LARS in Parkinson’s disease OFF.

Does reward sensitivity alter with Parkinson’s disease compared to controls or with apathy in Parkinson’s disease on this task? Taking into account all the data (control participants and Parkinson’s disease patients ON and OFF dopamine and drug-naïve) a linear mixed effects model was performed on average pupil reward sensitivity (difference between 0p and 50p reward level over the 1000 ms epoch) for each individual participant. This being the highest level of analysis, overall there was no difference between Parkinson’s disease patients and elderly controls [ *F* (1,96) = 0.02, *P =* 0.9]. There was, however, a main effect of apathy [ *F* (1,96) = 11.2, *P <* 0.01] with the apathetic Parkinson’s disease group demonstrating significantly reduced pupillary responses to reward than the non-apathetic group ( Fig. 4 A). Furthermore, while all comparisons between reward levels in the non-apathetic group were significant ( *P <* 0.003 between each reward magnitude), none were so in the apathetic group, indicating that pupillary response to reward is blunted in apathy.

There was also a main effect of drug on reward sensitivity [ *F* (1,96) = 10.6, *P <* 0.01] with reduced sensitivity when OFF, but no significant interaction between apathy and drug state. Planned Bonferroni corrected comparisons were also made. There were no overall significant differences between non-apathetic patients and elderly controls and also none overall between apathetic patients and elderly controls. However, when comparing elderly controls to apathetic cases OFF dopamine, apathetic patients with Parkinson’s disease were significantly less reward sensitive [ *F* (1,96) = 8.9, *P =* 0.03]. Non-apathetic patients OFF dopamine were no different from elderly controls, nor were apathetic ON. However, non-apathetic patients with Parkinson’s disease ON were significantly more reward sensitivity than elderly controls [ *F* (1,96) = 15.3, *P =* 0.001].

**Figure 5:**
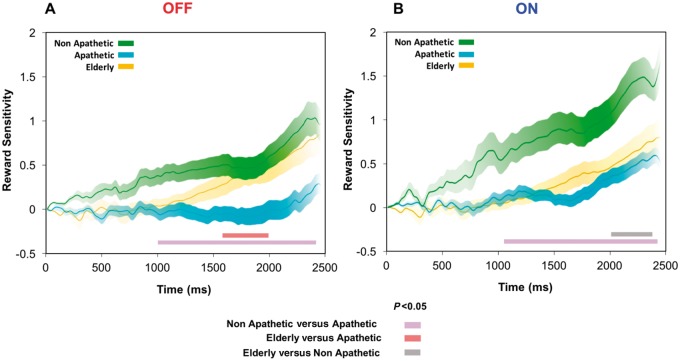
**Pupillary reward sensitivity in apathetic versus non-apathetic Parkinson’s disease patients, ON/OFF dopamine and elderly age-matched controls.** ( **A** ) OFF state. Mean pupil reward sensitivity (50p pupil change minus 0p pupil change) plotted over time between apathetic Parkinson’s disease patients (cyan) and non-apathetic Parkinson’s disease patients (green) when OFF dopamine or drug-naïve. A significant difference in sensitivity occurred from ∼1000 ms to the end of the trial, indicated by light purple bar ( *P* < 0.05). There was also a significant time period where apathetic patients had reduced reward sensitivity compared to elderly controls, indicated by light red bar. ( **B** ) ON State. Mean pupil reward sensitivity (50p pupil change minus 0p pupil change) plotted over time between apathetic Parkinson’s disease patients (cyan) and non-apathetic Parkinson’s disease patients (green). A significant difference in sensitivity occurred from ∼1100 ms to the end of the trial, indicated by light purple bar ( *P* < 0.05). There was also a significant time period where non-apathetic patients ON dopamine had increased reward sensitivity compared to elderly controls, indicated by grey bar.

These findings were also reflected in the time course of pupillary reward sensitivity as measured using multiple permutation testing to account for multiple comparison bias, with differences in pupillary response over time performed at each millisecond ( Fig. 5 ). When OFF dopamine, apathetic Parkinson’s disease patients had significantly less reward sensitivity compared to non-apathetic patients from ∼1000 ms to the end of each trial ( Fig. 5 A). They also showed some significant reduced reward sensitivity over time compared to elderly controls, but no differences were apparent between non-apathetic and elderly participants.

In the ON state, reward sensitivity increased over the trial in both apathetic and non-apathetic patients compared to when OFF ( Fig. 5 B). However apathetic patients remained significantly less sensitive than non-apathetic patients from ∼1100 ms even when ON dopamine. Indeed, when ON dopamine, apathetic patients rose to the same reward sensitivity level as controls, while the non-apathetic patients displayed, for a short time period, reward sensitivity that surpassed that of elderly controls. Although our groups are age-matched, we wished to ensure that age could not account for any of our effects. We therefore performed a repeat analysis with the effect of age regressed from our results. The outcomes reported above remained unchanged.

Next we analysed the results across all patients, rather than dichotomizing into apathetic and non-apathetic groups. A mixed ANCOVA was completed, with z-scored total LARS apathy score for each Parkinson’s disease patient used as a covariate. There was a significant main effect of drug state and of reward, an interaction between reward and apathy, as well as an interaction between drug state and reward [drug state *F* (1,28) = 18.3, *P <* 0.0001; reward *F* (1.4,39.0) = 15.9, *P <* 0.0001; apathy × reward *F* (1.4,39) = 5.1, *P <* 0.02; drug state × reward *F* (1.9,54.2) = 6.1, *P <* 0.005], but no significant three-way interaction between drug state, reward and apathy. Decomposing these results revealed that being ON dopamine led to a greater reward sensitivity and being apathetic was associated with reduced reward sensitivity.

To explore this effect further, a two-tailed Spearman’s Rank correlation between pupil reward sensitivity in ON and OFF state was performed as a function of total LARS score. A significant correlation was present both ON and OFF dopamine. The more apathetic a patient the less pupillary reward sensitivity they exhibited (Parkinson’s disease ON r _s_ = −0.56, *P <* 0.001, Fig. 4 B; Parkinson’s disease OFF r _s_ = −0.41, *P <* 0.01; Fig. 4 C). There was no significant difference in total levodopa equivalence doses between apathetic and non-apathetic groups **(**Table 1**)** .

Finally, comparisons were made to ensure that there were no differences in baseline pupil size in apathetic and non-apathetic Parkinson’s disease groups prior to hearing the reward on offer. Both ON and OFF dopamine, there was no significant difference between baseline pupil size across the two groups for any of the reward conditions, nor was there any significant interaction between reward and apathy group. Overall, these analyses show a blunting of pupil dilation by reward as a function of apathy, which cannot be accounted for by baseline pupil size.

#### Are pupil effects accounted for by generalized autonomic dysfunction?

One potential confound regarding any interpretation of reward sensitivity based on pupillary data is that they might reflect a generalized autonomic deficit, which potentially might be greater in apathetic Parkinson’s disease cases. To control for this we also examined heart rate variability obtained from a 30-min epoch when patients were asleep and completely at rest (as defined by no accelerometer activity detected during these periods), as in previous studies ( Haapaniemi *et al.* , 2001 ; Pospíšil *et al.* , 2008 ; Maetzler *et al.* , 2015 ). There were no significant differences between apathetic and non-apathetic Parkinson’s disease groups in any of the autonomic markers of heart rate variability that were analysed, including mean number of R waves; mean or standard deviation of interbeat interval; average root mean squared of the interbeat interval; high, low or very low frequency power spectral analysis; or low/high frequency ratio ( Supplementary Table 2 ).

### Saccadic eye movement measures

Our next stage of analysis was to examine differences in eye movement parameters between groups. The major focus of interest was peak velocity, a marker of invigoration of response which has been shown to be sensitive to rewards in both human and monkey studies ( Chen *et al.* , 2013 , 2014 ; Manohar *et al.* , 2015 ). We also analysed reaction times and saccadic variability, which are discussed later.

#### Peak velocity increases with incentive on offer

**Figure 6:**
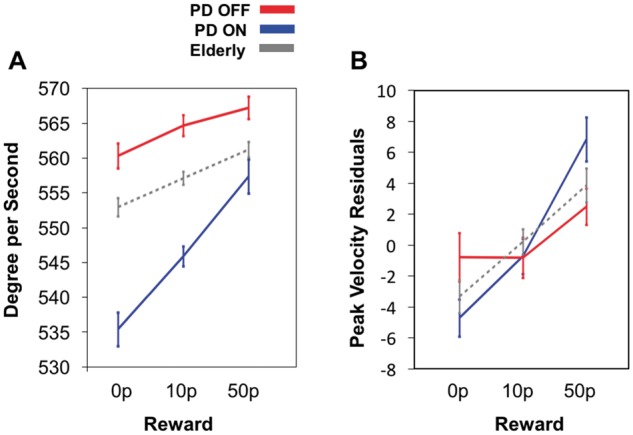
**Saccadic responses in patients and elderly controls.** ( **A** ) Average saccadic peak velocity in degrees per second as a function of reward for elderly controls (dashed grey), Parkinson’s disease ON (blue) and Parkinson’s disease OFF (red). Significantly steeper saccade reward sensitivity slopes were present in Parkinson’s disease ON compared to Parkinson’s disease OFF and elderly controls. There was increased invigoration of movements for bigger rewards in all groups, with increased incline when ON dopamine. ( **B** ) Visual representation of sensitivity slopes when changes in amplitude were accounted for by linear regression. Residual velocities plotted as a function of reward show that greater reward sensitivity is still present in Parkinson’s disease ON when compared to Parkinson’s disease OFF and compared to elderly participants at the largest level of reward. PD = Parkinson’s disease.

A repeated measures ANOVA performed on saccadic peak velocity for the three reward levels in young controls demonstrates a main effect of reward [ *F* (1.4,26.6) = 18.24, *P <* 0.001]. There was a significant increase between each reward level (all comparisons between rewards were significant, *P <* 0.01). This effect was also present in elderly controls [main effect of reward, *F* (1.7,52.0) = 9.2, *P <* 0.001]. Again, peak velocity significantly increased with each increment in reward (all comparisons *P <* 0.05; Fig. 6 A). It is well established that peak velocity scales with saccade amplitude—the so-called ‘main sequence’ ( Bahill *et al.* , 1975 ). It is possible that the increases in peak velocity we observed with reward reflect only increases in saccade amplitude: that eye movements become larger when more reward is on offer. To examine this issue, the amplitude of each saccade was factored out by performing a linear regression on the raw saccade data and obtaining residual velocities **(**Fig. 6 B). Sensitivity to reward remained even after factoring out amplitude changes for both controls and Parkinson’s disease patients, so the effect of incentives on peak velocity cannot be attributed to an invigoration of saccade length.

In the ON state, Parkinson’s disease patients showed greater reward sensitivity than when OFF ( Fig. 6 A). There was a significant main effect of both drug state and reward on peak velocity, as well as a drug state by reward interaction [drug state, *F* (1,28) = 6.0, *P <* 0.02; reward, *F* (1.6,44.9) = 14.9, *P <* 0.0001; drug state × reward, *F* (1.7,48.5) = 7.0, *P <* 0.003]. Of note, overall saccadic velocity was greater when OFF dopamine, despite having a reduced sensitivity to reward (shallower slope) compared to ON. This effect was due to larger amplitude saccades when OFF as the effect was abolished when amplitude is factored out ( Fig. 6 B). Pairwise comparisons showed a significant increase in saccadic peak velocity between every reward level in Parkinson’s disease ON (all reward level comparisons, *P <* 0.01). But in the OFF state there was only a significant difference between 0p versus 50p ( *P <* 0.04).

Next, saccadic reward sensitivity was calculated for each participant. This was taken as mean difference in peak velocity between the 50p and 0p conditions. A within subjects (drug state) and between subject (apathy/non-apathy group) ANOVA was performed. There were no significant effects of apathy, nor was there an interaction between drug state and apathy. There was however a main effect of drug state, with greater reward sensitivity in peak velocity when ON than OFF [ *F* (1,28) = 10.8, *P <* 0.003]. There was no significant correlation between UPDRS-III motor severity score and peak velocity reward sensitivity ON or OFF dopamine. These analyses, therefore, reveal that saccadic peak velocity scales with incentive on offer and is greater in the ON state but, unlike the pupillometry findings, there was no relationship to clinical apathy. Thus there is a dissociation between pupil and saccadic measures with respect to apathy.

Finally, we also compared the Parkinson’s disease group to elderly controls. In the ON state, patients appeared to have greater sensitivity to rewards compared to controls [main effect of reward *F* (1.6,93.4) = 26.8, *P <* 0.0001; reward × group interaction *F* (1.6,93.4) = 5.5, *P <* 0.01]. Pairwise comparisons in Parkinson’s disease ON revealed significantly greater increases in peak velocity at every increment in reward level ( *P <* 0.0001). Elderly controls, however, only showed a significant difference between the 0p versus 50p conditions ( *P <* 0.02); the remaining comparisons (0p versus 10p and 10p versus 50p) did not reach significance. Comparison of elderly controls to Parkinson’s disease OFF showed a significant main effect of reward [ *F* (1.9,110.4) = 10.0, *P <* 0.0001] but no significant interaction ( Fig. 6 A). To ensure that participants understood the task and were performing as instructed, saccadic peak velocity and saccadic amplitude variability were also used as an indicator of task performance. As demonstrated above, saccadic velocity scaled with larger incentives signifying participants understood that moving faster would result in obtaining larger rewards. Accuracy (as measured by saccadic variability) was also assessed. Increased accuracy in all groups for increasing reward magnitudes was observed ( Supplementary material ).

#### Saccadic reaction times, learning

In our task, reward did not significantly affect reaction time in any of the groups tested. To ensure the differential effects of reward observed in peak saccadic velocity and pupillary response were not attributable simply to faster learning of the task by particular groups or by being ON or OFF dopamine in Parkinson’s disease patients, the average reaction time for each reward level was compared for the first versus second half of the experiment. As reward obtained was dependent on reaction time, if learning was a significant factor in the task design then reaction time should decrease as participants learn that the faster they react, the larger the reward obtained. In young and elderly controls there was no significant difference between first and second halves of the experiment, nor an interaction between experiment half and reward. Parkinson’s disease patients, both ON and OFF dopamine or when broken down to apathetic and non-apathetic groups, displayed the same pattern of results, with no significant learning effect detected.

## Discussion

The findings presented here reveal that apathetic individuals with Parkinson’s disease exhibit significantly less pupillary response during the evaluation of reward compared to non-apathetic patients ( Fig. 5 ). Dopamine was found to play a key role in this valuation, with blunting of pupillary reward sensitivity when patients were OFF and restoration of sensitivity when ON dopaminergic medication ( Fig. 4 ). Although there were no significant associations between clinical apathy level and saccadic velocity, dopamine did influence oculomotor responses for rewards. Patients ON showed increased invigoration of saccadic velocity that scaled with reward magnitude, compared to when OFF ( Fig. 6 ). All these findings were independent of total dopamine equivalent dose, autonomic dysfunction and severity of clinical motor symptoms.

### Pupillary response sensitivity to reward

This study is the first to relate pupillary measures of reward sensitivity to clinical apathy. It demonstrates that reward insensitivity is dopamine-dependent and may contribute to disorders of motivation ( Robbins and Everitt, 1996 ; Sinha *et al.* , 2013 ). Pupillometry has been used to probe cognitive processes ( Wang and Munoz, 2015 ), with modulation of pupil size associated with a range of non-luminance dependent factors, including arousal, salience and reward ( Varazzani *et al.* , 2015 ; Joshi *et al.* , 2016 ). Various neurotransmitters are considered to be involved in this process, including dopamine ( Korczyn and Keren, 1980 ; Manohar and Husain, 2015 ) with potential interactions occurring between dopaminergic and noradrenergic systems ( Guiard *et al.* , 2008 ). Moreover, dopamine-related brain regions, including the basal ganglia, have been implicated in pupil size change ( Wang and Munoz, 2015 ).

As behavioural responses and pupil diameter are influenced by stimulus salience, we ensured that timings and auditory volume of all cues, stimulus luminance and reward levels were matched, leaving only individuals’ physiological response to the valuation of the rewards as the variable factor. Of course, larger rewards might be perceived as more salient and therefore incite greater arousal and increased pupillary change. This response would nevertheless still index the sensitivity that individuals have to each reward magnitude. In addition, increased arousal may place individuals in a heightened state ready to perform goal-directed actions ( Ressler, 2004 ). By this reasoning, reward sensitivity simply describes the degree to which incentives modulate physiological response (see Supplementary material for further discussion).

The findings presented here show that apathetic patients with Parkinson’s disease seemingly lose the ability to differentiate value of rewards of increasing magnitude, at least as indexed by pupillary reward sensitivity. Note though, that we would not argue that reward insensitivity completely accounts for apathy. Rather it may be one component of the syndrome, with apathy in Parkinson’s disease representing manifestations of an under-activated approach system ( Shulman, 2000 ) secondary to dysfunctional arousal mechanisms linking reward to motivation. It may be the case that apathetic patients need a greater amount of dopamine to normalize their clinical symptoms, over and beyond the level indexed by our pupillary reward measure. Indeed, patients with Parkinson’s disease in general might require greater reward sensitivity compared to controls to motivate themselves to perform a goal-directed task.

The exact neural pathways orchestrating pupillary and cognitive process, however, are not clearly defined. Locus coeruleus noradrenergic (LC-NA) projections may facilitate this connection via arousal ( Rajkowski *et al.* , 1994 ) or decision-making processes ( Nieuwenhuis *et al.* , 2005 ). Dopaminergic projections also share common connections to LC-NA pathways, so other systems are likely involved ( Wang and Munoz, 2015 ) with evidence for a dopamine and noradrenaline interaction ( Delaville *et al.* , 2011 ). Abnormal pupillary reward sensitivity in Parkinson’s disease patients with apathy may therefore arise through dysfunction of brainstem arousal processes, in which dopamine and noradrenaline interact to link reward to motivation ( Delaville *et al.* , 2011 ; Manohar and Husain, 2015 ).

Parkinson’s disease patients often suffer from executive deficits ( Dirnberger and Jahanshahi, 2013 ; Gratwicke *et al.* , 2015 ). The use of physiological measures like pupillometry to index value placed on monetary incentives minimizes executive function requirements ( Hong and Hikosaka, 2008 ; Chen *et al.* , 2014 ; Rudebeck *et al.* , 2014 ; Manohar and Husain, 2015 ; Manohar *et al.* , 2015 ). Our findings suggest that apathetic Parkinson’s disease patients place less value on rewards in the evaluation stage of goal-directed action. This may represent a reduction in the ‘wanting’ or motivation to obtain rewarding stimuli ( Berridge, 2007 ), and account for some of the manifestations of apathy such as reduced, self-initiated goal-directed behaviour.

It is important to ensure that any differences in pupillary response to reward between apathetic and non-apathetic Parkinson’s disease patients are not secondary to generalized systemic autonomic dysfunction. Investigations using ambulatory ECG recordings in Parkinson’s disease patients have revealed that autonomic dysfunction can be detected using measures of heart rate variability ( Haapaniemi *et al.* , 2001 ; Pospíšil *et al.* , 2008 ; Maetzler *et al.* , 2015 ). Using several different variability measures, we found that apathetic patients were not significantly different from non-apathetic patients. Thus the pupillary response differences to reward between the two groups were not associated with differences in more global autonomic dysfunction.

### Invigoration of saccadic velocity by reward

Within the Parkinson’s disease group, an interesting dissociation was found between the physiological changes in pupillary response to reward incentivization and the motor response performed to obtain it ( Niv *et al.* , 2007 ; Kurniawan *et al.* , 2010 , 2011 ). Thus although pupillary reward sensitivity was reduced in apathetic patients, they still maintained invigorated movement, indexed by peak saccadic velocity, when making an externally cued goal-directed action. One explanation might simply lie in a larger dynamic range for the pupillary measure in comparison to the saccade metric. Pupil modulation for reward in the time period of interest showed up to 59% increase between 0p and 50p levels in Parkinson’s disease patients ON dopamine, whereas saccadic differences were only of the order 3.9%, perhaps explaining why no association with apathy was detected.

Another possibility is that apathy in Parkinson’s disease is a disorder of intrinsic reward evaluation rather than specifically energization of motor actions, despite both seemingly being dopamine-dependent. In daily life, behavioural apathy is manifest as a reduction of self-initiated goal-directed behaviour. A common complaint of carers is that patients with behavioural apathy will perform actions if prompted, but not of their own volition. In the experiments conducted here, participants were cued by an extrinsic stimulus (target onset) to make an eye movement and their saccadic velocities scaled with the magnitude of incentives on offer. It is possible that while the reward evaluation system linked to pupillary change is dysfunctional in apathy, externally cued actions remain less affected than self-initiated ones. Thus when reward evaluation is used to guide self-generated actions there might be less invigoration of such behaviour compared to externally prompted ones. Intriguingly, a recent study has documented that pupillary dilation prior to making saccades that require greater voluntary control (anti-saccades) is blunted in Parkinson’s disease ( Wang *et al.* , 2016 ), although this investigation did not seek to explore an association with apathy.

A key deficit in Parkinson’s disease apathy may therefore be associated with ventral striatum, involved in encoding value of upcoming actions ( Rowe *et al.* , 2010 ), and medial frontal areas including supplementary and pre-supplementary motor cortex (SMA and pre-SMA) ( Nachev *et al.* , 2008 ). These medial frontal regions encode movements for reward in monkey single cell recordings ( Scangos and Stuphorn, 2010 ) and may be associated with self-initiated movements. They are particularly related to internally-driven motivational processes ( Bouret and Richmond, 2010 ) necessary for enhancing ventral striatum responses to the anticipation of reward ( Pujara *et al.* , 2016 ). In our apathetic cohort, the action initiation subscore of the LARS, which assesses voluntary action initiation, was significantly lower compared to non-apathetic patients with Parkinson’s disease. It has also been suggested that later onset Parkinson’s disease has greater loss of ventral striatal dopamine, vital for maintaining reward sensitivity ( Kish *et al.* , 1988 ; Sinha *et al.* , 2013 ). This is consistent with imaging findings that show ventral striatal atrophy in Parkinson’s disease cases with apathy ( Carriere *et al.* , 2014 ).

In our study, saccadic peak velocity sensitivity to reward was reduced in the Parkinson’s disease group overall when OFF dopamine compared to ON ( Fig. 6 ). Blunted action speed for rewards, as demonstrated by reduced saccadic peak velocities in dopamine-depleted states, may be representative of abnormal reward rate monitoring ( Niv *et al.* , 2007 ; Manohar *et al.* , 2015 ), perhaps reflective of reduced dopamine levels within the basal ganglia ( Levy and Dubois, 2006 ; Niv, 2013 ). Our task maintained a fixed reward rate using an adaptive staircase that was based on individual performance to keep the amount obtained equal for each participant. Parkinson’s disease patients ON dopamine appeared to be able to monitor the average rate of reward irrespective of apathy level, as demonstrated by increased saccadic vigour to maximise reward obtained, but their evaluation of this reward was disrupted in apathy, as indexed by the blunted pupillary response.

### Reward sensitivity and apathy in Parkinson’s disease

Other emerging evidence has suggested that impaired incentive processing may be contributory to apathy in Parkinson’s disease. A recent study examined feedback-related negativity responses to rewards ( Martinez-Horta *et al.* , 2014 ). Parkinson’s disease patients with apathy display reduced amplitude differences in feedback-related negativity signals while performing a gambling task, which may reflect compromised mesocorticolimbic reward pathways, and is consistent with the findings reported here. Indeed, apathy in Parkinson’s disease appears to be associated with nucleus accumbens atrophy ( Carriere *et al.* , 2014 ), an area implicated in reward evaluation ( Berridge and Kringelbach, 2008 ). Apathetic behaviours have also been reported in the 1-methyl-4-phenyl-1,2,3,6-tetrahydropyridine (MPTP) monkey model, where dopaminergic depletion is associated with ventral tegmental area as well as cortical disruption ( Tian *et al.* , 2015 ). Overall, dopamine pathway dysfunction in the nucleus accumbens and ventral tegmental area are reported to be the strongest predictors of apathy in this animal model of Parkinson’s disease ( Brown *et al.* , 2012 ).

The influence of dopamine on reward sensitivity is also apparent in other work that examines its role in reward and effort-based decision-making in Parkinson’s disease. ON dopaminergic medication, patients are more willing to exert effort for the same reward level compared to when OFF ( Chong *et al.* , 2015 ), perhaps via dopamine’s ability to reduce subjective effort costs and increase reward sensitivity. Similarly, reductions in dopamine therapy after implantation of STN-DBS for motor symptoms in Parkinson’s disease can potentially unmask existing mesolimbic pathway degeneration that leads to dopamine-sensitive apathy ( Thobois *et al.* , 2010 ).

In our study, there was no difference in the average levodopa daily dose between the apathetic and non-apathetic patients. One possible explanation for differences in reward sensitivity between the groups might be differential degeneration of ventral versus dorsal striatal regions. Later onset Parkinson’s disease may be associated with greater loss of ventral striatal dopamine ( Kish *et al.* , 1988 ; Sinha *et al.* , 2013 ) while in comparison, dorsal striatal function, crucial for motor control, is better preserved ( Tremblay *et al.* , 2015 ). Therefore, in later onset Parkinson’s disease, replacement of dopamine might still be insufficient to overcome apathy, despite improving motor symptoms. In our cohort, apathetic patients were on average 5 years older at diagnosis, consistent with this hypothesis.

Finally, the diminished response to dopaminergic medication in apathy also raises the possibility of dopamine resistance ( Carriere *et al.* , 2014 ) and the potential that other neurotransmitter pathways could play a role in the disorder ( Chaudhuri *et al.* , 2006 ). Although more work is needed to explore this further, a recent trial has reported effective treatment of apathy using the cholinesterase inhibitor, rivastigmine, in non-demented patients with Parkinson’s disease ( Devos *et al.* , 2014 ).

## Conclusion

This study demonstrates that reward sensitivity, modulated by dopamine, is blunted in patients with Parkinson’s disease suffering from clinical apathy. The findings raise the possibility that reward insensitivity may be a contributory mechanism to apathy and that it can be indexed through pupillary changes in response to monetary incentivization, independent of motor action. The results highlight how personalized physiological assessment in Parkinson’s disease can reveal potentially inadequate drug treatments of non-motor symptoms, and provide a basis for novel objective clinical markers of motivation in neurodegenerative conditions.

## Supplementary Material

Supplementary Data

## Abbreviations

LARS =: Lille Apathy Rating Scale
UPDRS =: Unified Parkinson’s Disease Rating Scale
