# Supplementary material for: Reward sensitivity deficits modulated by dopamine are associated with apathy in Parkinson’s disease
Source: Brain. 2016 Jul 24;139(10):2706–21. doi: 10.1093/brain/aww188 (PMC5035817; doi:10.1093/brain/aww188)
Supplement: Supplementary Data [file aww188_supplementary_data.zip › brain-2016-00419-File009.pdf]

Supplementary Methods | Muhammed *et al*

Methods:

Adjustable criteria were used which was based on the reaction time (RT) of the preceding 20 trials across all three reward conditions. RT, which was taken as the time from target onset until a saccade reached the target, was employed to calculate the reward. This was calculated to the nearest pence using the following:

$$R\ (t) = \ R_{max}\ \cdot \min\left(e^{\frac{\tau 2-1}{\tau 1}}, 1\right)$$

R is the reward obtained for the current trial. *t* is the time taken to reach the target. *Rmax* is the maximum reward value that could be won on a given trial.
$\tau 1$ and $\tau 2$ are adaptive reward criteria which were adjusted using the quantiles of the RT distribution for the preceding 20 trials. 10% of trials were kept faster than $\tau 1$ and 30% of trials slower than $\tau 2$. This adaptive process was irrespective of trial type and unknown to participants, it was designed to maintain constant and comparable reward rates across participants. This meant that any difference observed in performance between apathetic and non-apathetic groups could not be attributed simply to overall reward obtained.

Results:

<u>Saccadic amplitude variability</u>
A reduction in saccade amplitude variability was also apparent with increasing rewards on offer. This demonstrates both an increase in accuracy as well as velocity when larger rewards were on offer.

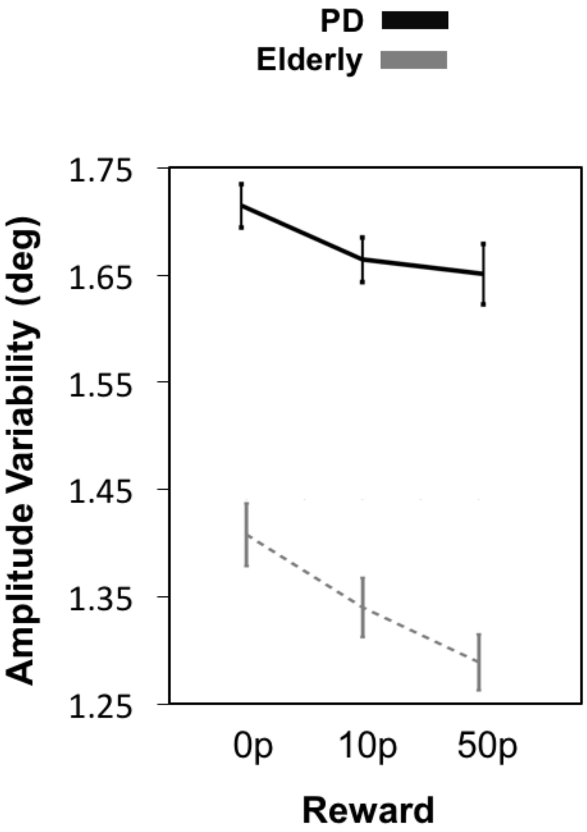

Figure 7. Saccade amplitude variability for PD (ON and OFF) compared to elderly controls. There was a main effect of group ($F(1,30) = 15.6$, $p<0.0001$) with PD (ON and OFF) being significantly more variable compared to elderly controls. There was also a main effect of reward with higher rewards resulting in less variable saccades ($F(2,60) = 6.3$, $p <0.01$). No significant interaction was present.

<u>Questionnaire correlations</u>

There were no correlations with pupillary or saccadic reward sensitivity and cognitive function as measured using the MoCA. This was true both in the ON and OFF state and for all of the individual MoCA sub-components which comprise fluency, trial making and assessments of attention, language and memory. The same was also true for depression scores using the BDI-II (all $p>0.05$).

Heart Rate Variability

	Non Apathetic Patients	Apathetic Patients	p-value
Mean Number of R Waves	1835 (237)	1774 (559)	0.65
Mean interbeat interval (seconds)	0.99 (0.14)	0.98 (0.15)	0.99
Mean standard deviation of interbeat interval	0.06 (0.05)	0.04 (0.03)	0.10
Root mean squared of interbeat interval (RMSSD)	0.07 (0.08)	0.04 (0.03)	0.65
Mean high frequency (HF) (ms^2)	149.8 (139)	114.9 (137)	0.29
Mean low frequency (LF) (ms^2)	199.7 (195)	153.9 (171)	0.25
Mean low frequency/High frequency ratio	2.83 (4.3)	1.89 (2.1)	0.84
Mean very low frequency (VLF) (ms^2)	161.9 (153)	180 (215)	0.44

Table 2. Heart rate variability in apathetic vs non-apathetic PD cases
Heart rate variability data comparing apathetic and non-apathetic PD patients. Numbers in brackets represent standard deviations.

Discussion:

To clarify our view on the definition of reward sensitivity. We take "reward sensitivity" to be an empirical construct that describes the degree to which incentives modulate physiological response. Parkinson's disease patients with apathy on clinical questionnaire assessment may show less pupillary modulation by reward for several reasons: reduced attention to the incentives, reduced overall motivation or maximal energy output, or higher cognitive costs for exerting effort or processing the cue. On our broad view, attentional costs may be part of apathy and motivation in general. Therefore, if an individual's behaviour is less affected by reward for any reason, for example because they are less alert or attentive, this would still count as contributing to reduced reward sensitivity, and inform us of their motivational state.
